# Synthesis of Cationized Magnetoferritin for Ultra-fast Magnetization of Cells

**DOI:** 10.3791/54785

**Published:** 2016-12-13

**Authors:** Sara Correia Carreira, James P.K. Armstrong, Mitsuhiro Okuda, Annela M. Seddon, Adam W. Perriman, Walther Schwarzacher

**Affiliations:** ^1^Bristol Centre for Functional Nanomaterials, University of Bristol; ^2^Department of Materials, Imperial College London; ^3^Self Assembly Group, CIC nanoGUNE; ^4^Ikebasque, Basque Foundation for Science; ^5^School of Cellular and Molecular Medicine, University of Bristol; ^6^H.H. Wills Physics Laboratory, University of Bristol

**Keywords:** Bioengineering, Issue 118, magnetoferritin, magnetic nanoparticles, protein cage, cationization, surface functionalization, magnetic labeling, magnetic cell separation, stem cells

## Abstract

Many important biomedical applications, such as cell imaging and remote manipulation, can be achieved by labeling cells with superparamagnetic iron oxide nanoparticles (SPIONs). Achieving sufficient cellular uptake of SPIONs is a challenge that has traditionally been met by exposing cells to elevated concentrations of SPIONs or by prolonging exposure times (up to 72 hr). However, these strategies are likely to mediate toxicity. Here, we present the synthesis of the protein-based SPION magnetoferritin as well as a facile surface functionalization protocol that enables rapid cell magnetization using low exposure concentrations. The SPION core of magnetoferritin consists of cobalt-doped iron oxide with an average particle diameter of 8.2 nm mineralized inside the cavity of horse spleen apo-ferritin. Chemical cationization of magnetoferritin produced a novel, highly membrane-active SPION that magnetized human mesenchymal stem cells (hMSCs) using incubation times as short as one minute and iron concentrations as lows as 0.2 mM.

**Figure:**
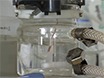


## Introduction

Surface binding or internalization of superparamagnetic iron oxide nanoparticles (SPIONs) has enabled magnetization of a variety of cell types for applications such as imaging and remote manipulation.^1^ However, achieving sufficient cellular magnetization can be a challenge, particularly when the interaction between the SPION and the cell surface is weak.^2^ In the past, prolonged exposure or high SPION concentrations have been employed as strategies to overcome this challenge.^3,4^ Nevertheless, these strategies are problematic because they increase toxicity^5,6^ and have very limited success in cell types with low internalization rates, such as lymphocytes.^7 ^To enhance cellular uptake of SPIONs, several surface functionalization approaches have been explored. For instance, antibodies have been used to promote receptor-mediated endocytosis,^8^ while non-specific uptake can be achieved using transfection agents^9,10^ or cell-penetrating species, such as HIV tat-peptide.^11,12^ However, antibody and peptide functionalization approaches are limited by expensive reagents and complex synthetic preparation, while transfection agents can induce nanoparticle precipitation and adversely affect cell function.^13,14 ^

We recently reported the synthesis of chemically cationized magnetoferritin, a novel SPION which was highly effective in magnetizing human mesenchymal stem cells (hMSCs) using incubation times as short as one minute.^15^ Magnetoferritin is synthesized by reconstituting a SPION inside the de-mineralized cavity of the iron storage protein ferritin.^16^ This protein-based SPION combines many properties that make it well suited for cell magnetization, such as control over the magnetic properties of the magnetic core,^17-19^ and biocompatibility and aqueous solubility conferred by the protein shell. Furthermore, surface functionalization is easily achieved due to addressable amino acids that can be chemically^20-22^ or genetically modified.^23-25^ We have shown that chemical cationization of the acidic amino acid residues of the protein shell generates a stable nanoparticle that readily interacted with anionic domains on the cell surface leading to rapid and persistent cell magnetization. This procedure eliminates the need for laborious functionalization and lengthy incubation protocols, and due the non-specific labelling mechanism this rapid magnetization strategy should find wide-spread application in other cell types. Here, we present an in depth report of the ultra-fast cell labeling method, including detailed protocols of the synthesis, purification and surface functionalization of cationized magnetoferritin.

## Protocol

Human mesenchymal stem cells (hMSC) were harvested from the proximal femur bone marrow of osteoarthritic patients undergoing total hip replacement surgery, in full accordance with Bristol Southmead Hospital Research Ethics Committee guidelines (reference #078/01) and after patient consent was obtained.

### 1. Magnetoferritin Synthesis and Purification

1. **General remarks**
  1. Perform magnetoferritin synthesis in a sealable, double-jacketed reaction vessel at 65 °C under nitrogen atmosphere to restrict oxidation of the metal precursors. Inject metal salt solutions and hydrogen peroxide through access ports in the lid into the reaction vessel using a syringe pump.
  2. After magnetoferritin synthesis, purify the protein by anion-exchange chromatography (see section 1.4) to remove nanoparticles not enclosed in the protein cavity, followed by size-exclusion chromatography (see section 1.5) to isolate protein monomers. Determine protein concentration using a Bradford assay (see section 1.6).
2. **Preparations prior to synthesis**
  1. Deoxygenate 500 ml of deionized water by placing a tube connected to a nitrogen gas cylinder in the water, sealing the vessel with cling film and bubbling through nitrogen gas for approximately 60 min.
  2. Heat a water bath connected to the double jacketed reaction vessel to 65 °C. Add 75 ml of 50 mM 4-(2-hydroxyethyl)-1-piperazineethanesulfonic acid (HEPES) buffer (pH 8.6) into the reaction vessel, seal the vessel, and deoxygenate by bubbling nitrogen gas through the buffer solution for approximately 20 min. At the same time, stir the buffer solution using a magnetic stirrer.
  3. After deoxygenating the HEPES buffer solution, remove the tube supplying the nitrogen gas from the buffer and keep it suspended in the vessel to maintain a nitrogen atmosphere. Add apo-ferritin to achieve a final concentration of 3 mg/ml. Continue the magnetic stirring, but reduce the stirring speed if foaming occurs.
  4. Prepare a 25 mM stock solution of cobalt sulfate heptahydrate by dissolving 0.19 g in 50 ml of deoxygenated deionized water.
  5. Prepare a 25 mM solution of ammonium iron sulfate hexahydrate solution by dissolving 0.98 g in 100 ml of deoxygenated deionized water.
  6. Remove 2.5 ml of the ammonium iron sulfate hexahydrate solution and replace with 2.5 ml of the cobalt sulfate heptahydrate.
  7. Prepare an 8.33 mM solution of hydrogen peroxide in deoxygenated deionized water by first adding 965 μl of a hydrogen peroxide solution (30% w/w) to 9 ml of deoxygenated deionized water, and then adding 1 ml of this sub-stock to 99 ml of deoxygenated deionized water.
3. **Magnetoferritin synthesis**
  1. Inject 10.1 ml of both the iron-cobalt precursor and the hydrogen peroxide simultaneously into the apo-ferritin solution (magnetically stirred) at a flow rate of 0.15 ml/min using two syringe pump (total volume injected: 20.2 ml).
  2. During the injection period, deoxygenate another 500 ml of deionized water. NOTE: The contents of the reaction vessel gradually adopt a dark brown color as the reaction proceeds.
  3. Shortly before completion of the first injection, prepare another 100 ml of iron-cobalt precursor and hydrogen peroxide solution using the freshly deoxygenated deionized water.
  4. Repeat the injection step described in 1.3.1 using freshly prepared solutions of iron-cobalt and hydrogen peroxide.
  5. During the injection period, deoxygenate another 500 ml of deionized water, and proceed as described in 1.3.3 and 1.3.4.
  6. After the third injection, leave the solution to mature for 15 min under stirring.
  7. Add 1.5 ml of a 1 M sodium citrate solution to the reaction vessel to chelate free metal ions in solution.
  8. Remove the solution from the reaction vessel into 50 ml centrifuge tubes, centrifuge for 30 min at 4,350 x g and pass the supernatant through a 0.22 µm syringe filter. At this stage, the filtered supernatant can be stored at 4 °C until purification.
4. **Ion exchange chromatography**
  1. Load the sample onto a column (2.5 cm diameter, 20 cm long) containing a cationic matrix using a peristaltic pump at a flow rate of 10 ml/min.
  2. Wash the column with approximately 100 ml of running buffer (50 mM Tris(hydroxymethyl)aminomethane (Tris) buffer, pH 8.0) using a gradient pump at a flow rate of 10 ml/min.
  3. To elute the protein, wash the column at 10 ml/min with increasing concentrations of sodium chloride (NaCl) in Tris buffer: 150, 500 and 1,000 mM final NaCl concentration, 150 ml of each concentration.
  4. As the protein elutes at a NaCl concentration of 500 mM, collect it in 50 ml fractions using an automated fraction collector. NOTE: For a detailed protocol of ion exchange chromatography consult previously published protocols.^26^
5. **Size exclusion chromatography**
  1. Concentrate the 150 ml of magnetoferritin to a volume of approximately 2 ml using a 15 ml centrifugal filter unit followed by a 4 ml volume unit. Refer to the manufacturer's instructions of the centrifugal filter units (see material list) for a detailed protocol of this procedure.
  2. Load the concentrated sample onto a gel filtration column using an injection loop.
  3. Wash the column with running buffer (50 mM Tris buffer, pH 8.0, containing 150 mM NaCl) at a flow rate of 1.3 ml/min.
  4. Collect 6 ml fractions using an automated fraction collector. Protein monomers elute last. At this point, purified magnetoferritin can be stored at 4 °C until cationization. NOTE: For a detailed protocol of size exclusion chromatography using a gel filtration column consult previously published protocols.^27^
6. **Determining protein concentration**
  1. Prepare ferritin standards of 0.06, 0.125, 0.25, 0.5 and 1 mg/ml in 50 mM Tris buffer using commercially available horse spleen ferritin. NOTE: The protein concentration of commercially available ferritin is stated on the bottle. If not, contact the supplier for this information.
  2. Dilute magnetoferritin samples of unknown concentration in 50 mM Tris buffer until the color of the solution approximately matches the color of the 0.5 mg/ml standard. Make a note of the dilution factor.
  3. Add 10 μl of each standard and magnetoferritin sample in triplicate into wells of a 96 well plate.
  4. Add 200 μl of ready-made Bradford reagent to each well (refer to materials list for Bradford assay reagent details).
  5. Incubate at room temperature for 8 minutes.
  6. Measure the absorbance at *λ *= 595 nm using a microplate reader.
  7. Plot the mean absorbance of the ferritin standards as a function of protein concentration and use the slope and intercept of the linear fit to calculate the concentration of the magnetoferritin sample. NOTE: Take into account the dilution factor that was used to adjust the magnetoferritin sample to the standard curve.

### 2. Magnetoferritin Cationization

1. **General remarks** NOTE: For magnetoferritin cationization, *N,N*-dimethyl-1,3-propanediamine (DMPA) was coupled to aspartic and glutamic acid residues on the MF surface using *N*-(3-dimethylaminopropyl)-*N*'-ethylcarbodiimide hydrochloride (EDC).
  1. Carry out the reaction at room temperature under stirring. The protocol given below is for the cationization of **10 mg of magnetoferritin in a total volume of 5 ml **(protein concentration 2 mg/ml). However, scale up or down by keeping the protein:DMPA:EDC ratios consistent, as well as the volumes of the buffer and magnetoferritin solution.
  2. Prior to the cationization reaction, dialyze the magnetoferritin samples into 50 mM phosphate buffer (pH 7) containing 50 mM NaCl. This is important to remove Tris elution buffer and avoid unwanted reactions between the Tris amine and the EDC-activated carboxylic acid residues. Determine the protein concentration after dialysis and adjust it to 4 mg/ml prior to cationization.
2. **Cationization protocol**
  1. Weigh out 374 mg of DMPA and dissolve in 2.5 ml of 200 mM 2-(*N*-morpholino)ethanesulfonic acid (MES) buffer.
  2. Adjust the solution pH to approximately 7 using concentrated (6 M) hydrochloric acid (HCl). **CAUTION: Perform this step in a fume hood, as addition of the acid to DMPA releases toxic fumes!**
  3. Add 2.5 ml of magnetoferritin solution at 4 mg/ml (final concentration of MES is 100 mM, final concentration of magnetoferritin is 2 mg/ml, total amount of protein is 10 mg).
  4. Add a magnetic stirrer and stir for 2 hr to equilibrate.
  5. Carefully adjust the solution to pH 5.0 using 1 M HCl.
  6. Add 141 mg of EDC powder to the DMPA/magnetoferritin solution.
  7. Continue stirring for 3.5 hr.
  8. Filter the solution through a 0.22 µm syringe filter to remove any precipitates.
  9. Add the solution to dialysis tubing with a 12-14 kDa molecular weight cut-off and dialyze against 4 L of 50 mM phosphate buffer (pH 7) containing 50 mM NaCl for 2 days at 4 °C, replacing the dialysis buffer at least three times during that period. NOTE: At this point, cationized magnetoferritin can be stored at 4 °C until further use.

### 3. Human Mesenchymal Stem Cell Labeling with Cationized Magnetoferritin

1. **General remarks**
  1. Perform all cell culture using class II laminar flow cabinets and humidified incubators at 37 °C and 5% carbon dioxide atmosphere.
  2. Culture cells as monolayers using 175 cm^2^ flasks and 20 ml of culture medium, which was replenished every 3 - 4 days. Culture medium consisted of Dulbecco's Modified Eagle's Medium (DMEM), containing 1,000 mg/L glucose, 10% (v/v) fetal bovine serum, 1% (v/v) penicillin/streptomycin solution, 1% (v/v) L-alanyl-L-glutamine solution and 5 ng/ml freshly supplemented human fibroblast growth factor.
2. **Magnetic labeling of hMSC with cationized magnetoferritin**
  1. Seed 150,000 hMSCs into 25 cm^2^ flasks and leave to adhere overnight at 37 °C.
  2. Filter sterilize cationized magnetoferritin through a 0.22 µm syringe filter and determine the protein concentration.
  3. Keeping sterile conditions, prepare a 0.5 μM solution of cationized magnetoferritin in phosphate buffered saline (PBS). Keep capped in a sterile culture hood until use.
  4. Wash the plated cells with 2 ml of room temperature PBS.
  5. Add 1 ml of the sterilized cationized magnetoferritin solution to the plated cells and incubate for the desired time period (1 min to 1 hr).
  6. Wash cells with PBS, and then harvest cells by adding 0.5 ml of trypsin/ethylenediaminetetraacetic acid (EDTA) and incubating at 37 °C for 5 min.
  7. Add 1 ml of culture medium to inactivate the trypsin/EDTA, transfer the solution to a 15 ml centrifuge tube and centrifuge for 5 min at 524 x g.
  8. Discard the supernatant and re-suspend the cell pellet in 0.5 ml magnetic separation buffer, consisting of 0.5% (w/v) fetal bovine serum and 2 mM EDTA in PBS.
3. **Magnetic cell separation**
  1. Attach the magnet to the multi stand, and add a magnetic separation column to the magnet. Place a pre-separation filter on the column.
  2. Place 0.5 ml of magnetic separation buffer in the pre-separation filter and let it run through both the filter and the column to wash and wet them.
  3. Place a 15 ml centrifuge tube under the column.
  4. Add 0.5 ml of the cell suspension in the filter reservoir of the magnetic separation column.
  5. When the reservoir is empty, add 0.5 ml of magnetic separation buffer.
  6. When the reservoir is empty, add further 0.5 ml of magnetic separation buffer.
  7. Repeat one more time (the total volume of magnetic separation buffer used for washing should be 1.5 ml). This wash step elutes all non-magnetized cells from the column (non-magnetized cell fraction).
  8. Remove the column from the magnet and place it in a fresh 15 ml centrifuge tube. Remove filter from the column reservoir.
  9. Add 1 ml of magnetic separation buffer to the reservoir and immediately push through the column using the plunger supplied by the manufacturer. This elutes the magnetized cells from the column into the centrifuge tube (magnetized cell fraction).
  10. Centrifuge both tubes for 5 min at 524 x g.
  11. Discard the supernatant and re-suspend the cell pellets in 0.3 ml PBS.
  12. Count the number of cells numbers in each fraction using a hemocytometer.
  13. Determine the fraction of magnetized cells, *M(%)*, using the following equation: 
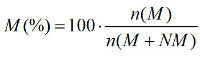
  where *n(M)* is the number of cells in the magnetized fraction and *n(M+NM)* is the sum of the number of cells in the magnetized and non-magnetized fractions.
4. **Preparing hMSCs labeled with cationized magnetoferritin for iron quantification using inductively coupled plasma optical emission spectroscopy (ICP-OES)** NOTE: The protocol may need to be adapted for other ICP-OES instruments. It is advisable to consult with the responsible technician.
  1. Centrifuge a known number of cells for 5 min at 524 x g.
  2. Remove the PBS supernatant and add 0.25 ml of 50% (v/v) nitric acid.
  3. Vortex mix the acidified cell suspension.
  4. Incubate at room temperature overnight.
  5. Add 4.75 ml of distilled water to each sample and vortex mix.
  6. Analyze with ICP-OES.^28^
  7. Normalize the measured iron content to the number of cells to determine a value for cellular iron content.

## Representative Results

TEM was used to confirm nanoparticle mineralization inside the apoferritin cavity and determine the average core size (**Figures 1A **and** 1B**). Image analysis of unstained magnetoferritin samples gave an average core diameter of 8.2 ± 0.7 nm, and aurothioglucose stain confirmed the presence of nanoparticles within the protein cage. Note that the images show a magnetoferritin sample that was further purified using magnetic separation to isolate uniform nanoparticle cores. Magnetoferritin samples that were not magnetically purified have a slightly broader core size distribution.^29^ Analysis of the structure of the magnetoferritin core using selected-area electron diffraction indicated the possible presence of the inverse spinel structure based on magnetite (Fe_3_O_4_) and/or maghemite (γ-Fe_2_O_3_), as well as the spinel structure due to Co_3_O_4_. Furthermore, Raman spectra revealed peaks attributed to Fe_3_O_4_, small amounts of γ-Fe_2_O_3_, and a cobalt ferrite (**Figure 1 C**). ICP-OES analysis of magnetoferritin showed an average of 102 μg of iron and 0.9 μg of cobalt per milligram of magnetoferritin.

A schematic is included, illustrating the subsequent cationization step (**Figure 2 A**). The hydrodynamic diameter of magnetoferritin and cationized magnetoferritin was 11.8 ± 1.1 nm and 12.5 ± 1.4 nm, respectively, as determined by dynamic light scattering. The cationization efficiency of covalent DMPA-coupling to magnetoferritin was assessed using zeta potentiometry and matrix-assisted laser desorption ionization time-of-flight (MALDI-TOF) mass spectrometry. The zeta potential changed from -10.5 mV for MF to + 8.3 mV for cationized magnetoferritin, confirming a change in surface potential from negative to positive (**Table 1**). Mass spectrometry experiments found a subunit molecular weight of 20.1 kDa for native apo-ferritin and 21.1 kDa for cationized apo-ferritin (**Figure 2 B**). This mass increase corresponds to approximately 12 coupled DMPA molecules per protein subunit, and the cationization of 288 residues on the entire 24-subunit protein.

Magnetic saturation and susceptibility were measured using SQUID magnetometry, and transverse and longitudinal relaxivity were measured using magnetic resonance imaging. Magnetic properties were similar for magnetoferritin and cationized magnetoferritin, indicating that cationization had negligible impact on the magnetic properties of the enclosed SPION (**Table 1**). Furthermore, these properties are similar to other iron oxide based nanoparticles,^19,30^ demonstrating that cationized magnetoferritin would be as suitable as conventional SPION-based MRI contrast agents in enhancing imaging contrast.

After a 30-minute exposure, the cell surface was densely covered with cationized magnetoferritin (**Figure 3 A**). However, after one week, no nanoparticles were found on the cell surface (**Figure 3 B**). Cationized magnetoferritin was remarkably effective at magnetically labelling hMSCs. Notably, exposing the cells to cationized magnetoferritin for one minute resulted in the magnetization of 92% of the cell population and the delivery of 3.6 pg of iron per cell. Increasing the incubation time to 15 minutes resulted in the magnetization of the entire cell population (**Figure 3 C**).


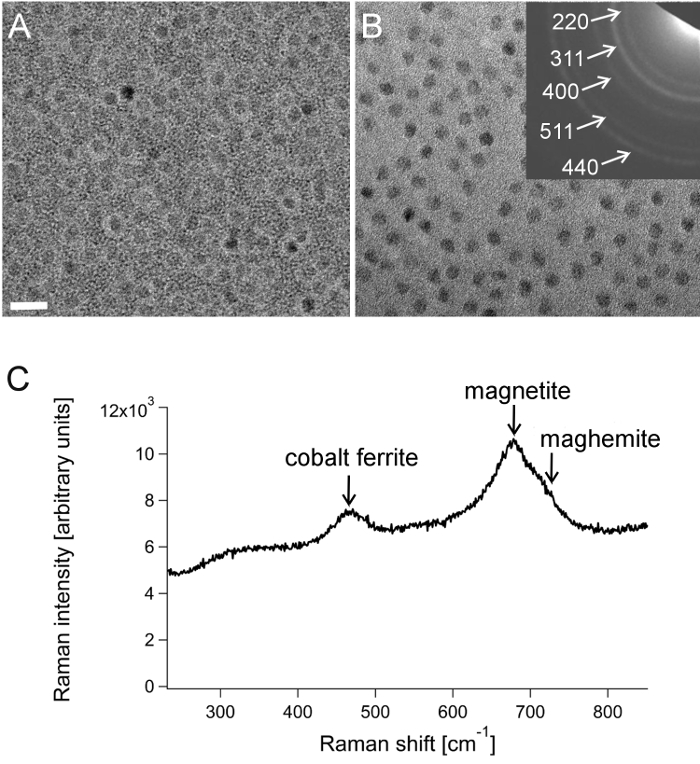
**Figure 1:Characterization of magnetoferritin cores doped with 5% cobalt.** TEM images of magnetoferritin stained with aurothioglucose (**A**) and unstained (**B**). Inset shows corresponding electron diffraction with magnetite indices. Scale bar: 20 nm. (**C**) Raman spectrum for magnetoferritin. The arrows indicate the main Raman vibration modes for cobalt ferrite (T_2g_), magnetite and maghemite (both A_1g_).^31,32^ The laser wavelength used was 532 nm. (Image adapted from Okuda *et al.^18^*). Note that this magnetoferritin sample had been further purified using magnetic separation, which isolated uniformly loaded magnetoferritin particles. Please click here to view a larger version of this figure.


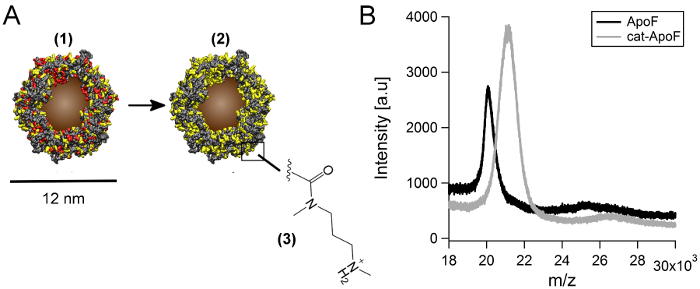
**Figure 2:Cationization of magnetoferritin. A**) Solvent accessible surface area representations showing the distribution of acidic (red) and basic (yellow) amino acid residues on the protein surface. Magnetoferritin (1) is modified to cationized magnetoferritin (2) by carbodiimide-mediated crosslinking of DMPA to acidic amino acid residues on the protein surface (3). **B**) Mass spectrometry analysis of apo-ferritin and cationized apo-ferritin subunits. Mass-to-charge (m/z) spectrum of apoferritin (ApoF) and cationized apoferritin (cat-ApoF) generated by MALDI-TOF. A mass increase from 20.1 kDa to 21.1 kDa is observed after cationization. (Image adapted from Correia Carreira *et al*.^15^) Please click here to view a larger version of this figure.


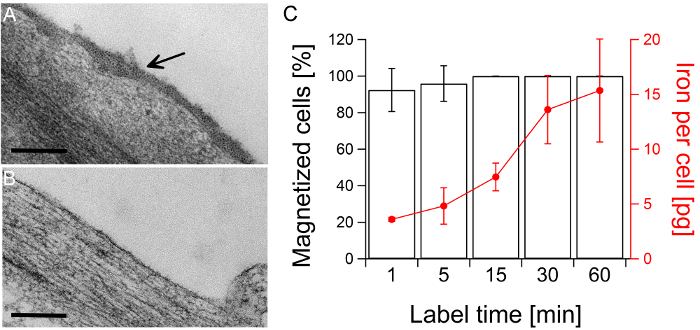
**Figure 3:Magnetic labeling and cell separation of hMSCs incubated with cationized magnetoferritin.A**) TEM image of hMSCs after a 30-minute incubation with cationized magnetoferritin. The arrow indicates the presence of magnetoferritin cores densely packed on the cell surface. Scale bars: 200 nm. **B**) TEM image of hMSC one week after labeling. The cell surface is clear of cationized magnetoferritin. **C**) Investigating the rapidity of magnetic labeling: 92% of the cell population were magnetized after a one-minute exposure with 0.5 μM cationized magnetoferritin, and the entire cell population was magnetized within 15 minutes. Iron content per cell was determined using ICP-OES. Average and standard deviation from three biological replicates are shown. (Image adapted from Correia Carreira *et al.^15^*) Please click here to view a larger version of this figure.

**Table:** 

	**MF**	**cat-MF**
**Hydrodynamic diameter [nm]**	11.8 ± 1.1	12.5 ± 1.4
**Zeta potential [mv]**	(-)10.4 ± 0.2	8.3 ± 0.7
**Magnetic saturation moment [Am^2^ kg^-1^]**	54.9 ± 1.6	55.3 ± 1.4
**Mass susceptibility [x 10^4^ m^3^ kg^-1^]**	1.75 ± 0.08	1.75± 0.07
**Longitudinal relaxivity [mM^-1^ sec^-1^]**	2.6 ± 0.1	2.3 ± 0.1
**Transverse relaxivity [mM^-1^ sec^-1^]**	44.6 ± 1.0	52.8 ± 0.8

**Table 1: Physicochemical characterization of magnetoferritin (MF) and cationized magnetoferritin (cat-MF).** (Table adapted from Correia Carreira *et al.^15^*)

## Discussion

TEM of magnetoferritin samples stained with aurothioglucose revealed the successful mineralization of nanoparticles inside the protein cage. Electron diffraction and Raman analysis of the nanoparticle core indicated the presence of a cobalt ferrite, indicating successful doping of the nanoparticle core with cobalt. This demonstrates that mixed-oxide nanoparticles can successfully be mineralized within the apo-ferritin cavity. Furthermore, we have shown previously that cobalt doping can be varied by altering the amount of cobalt precursor added to the reaction mixture, which enables tuning of the magnetic properties.^18^

Magnetoferritin synthesis can be performed in a variety of vessels, as long as they are tightly sealable and have access ports through which reactants can be introduced (*e.g.*, a three-neck round bottom flask). The reaction temperature should be maintained at 65 °C either by placing the vessel in a water/oil bath or using a double-jacketed vessel. Here, we used a double-jacketed electrochemical cell setup to perform the synthesis. To guarantee successful synthesis, maintaining the correct pH and avoiding oxygen contamination of the aqueous solutions is crucial. Metal salt solutions should always be prepared freshly prior to use rather than in advance. Furthermore, commercial apoferritin solutions can vary in quality and affect synthesis outcome (*e.g*., size of nanoparticle core mineralized). It can help to dialyze the apoferritin solution into 50 mM HEPES buffer (pH 8.6) prior to synthesis to remove any residual reducing agent used by the manufacturer. It is useful to make a note of the batch number of the apo-ferritin solution used for synthesis, so it can be specifically requested from the manufacturer should additional material need to be purchased. Furthermore, the protein concentration of commercially available apo-ferritin should be stated on the bottle, which can be used to calculate the volume of apo-ferritin solution needed for synthesis. If this is not the case, contact the supplier for this information.

The advantage of gradual addition of metal salts and hydrogen peroxide — as presented here and in previous reports — is that mineralization of the nanoparticle core can be controlled such that different loading factors (*i.e.,* nanoparticle sizes) can be achieved.^33^ Furthermore, it is possible to purify magnetoferritin further using a magnetic separation column, *e.g.,* a column packed with stainless steel powder secured inside an electromagnet.^34^ Thus, highly monodisperse nanoparticle cores can be isolated from the bulk magnetoferritin sample. However, for magnetic cell labelling as presented here this is not required. A limitation of magnetoferritin synthesis is the relatively low synthesis yield of about 10%, and the relatively high cost of commercial apo-ferritin solutions. However, apo-ferritin may also be prepared from cheaply available horse spleen ferritin by following established de-mineralization protocols.^16^

Cationization of magnetoferritin was achieved by adding a molar ratio of 250 molecules of DMPA and 50 molecules of EDC per negatively charged residue (calculations based on the amino acid sequence of horse spleen ferritin). This excess of reagent over protein resulted in high cationization efficiencies, comparable also to previously reported results for the cationization of ferritin.^35^ For MALDI-TOF analysis, apoferritin and cationized apoferritin were used because of the excessive molecular mass of the magnetoferritin core. To yield high cationization efficiencies, optimal pH is also crucial. EDC-mediated crosslinking is most effective under mildly acidic conditions, and we found that pH 5 yielded optimal cationization results for magnetoferritin. However, for other proteins cationization pH may need to be optimized. Cationization at or close to the isoelectric point of the protein should be avoided, because this may lead to severe precipitation.

Stem cell magnetization with cationized magnetoferritin was highly efficient and could be achieved using incubation times well below 30 minutes. Even a one-minute incubation resulted in a cellular iron content of 3.6 pg, which is within the reported range required to influence T2 and T2* contrast for MRI.^36,37^ It is also remarkable that this efficient labeling is achieved with low extracellular iron concentrations. For example, previous studies using anionic nanoparticles report iron levels of 10 pg per cell after a 30-minute incubation period with 5 mM iron.^38^ In comparison, incubation with a cationized magnetoferritin solution containing 0.5 μM protein corresponds to incubation with approximately 0.2 mM iron and also yields approximately 10 pg of iron per cell after 30 minutes. We were not able to clearly identify any endocytotic vesicles using TEM. However, previous studies using cationized ferritin found that internalization occurred within the first ten minutes of exposure.^39,40^ Cationized ferritin could be localized in coated vesicles, indicating clathrin- or caveolin-dependent endocytosis. The same studies also reported that after 30 minutes of incubation, cationized ferritin was still present on the cell surface, as well as in multivesicular bodies, resembling lysosomes.

Further applications could include cationization of apo-ferritin cages loaded with other nanoparticles and/or functional molecules, such as anti-cancer drugs^41^ or quantum dots^42^. Cationization of these ferritin constructs could result in faster and more efficient delivery of their cargo to cells.

## Disclosures

The authors have nothing to disclose.
